# Estimation of D-Arabinose by Gas Chromatography/Mass Spectrometry as Surrogate for Mycobacterial Lipoarabinomannan in Human Urine

**DOI:** 10.1371/journal.pone.0144088

**Published:** 2015-12-03

**Authors:** Prithwiraj De, Anita G. Amin, Eloise Valli, Mark D. Perkins, Michael McNeil, Delphi Chatterjee

**Affiliations:** 1 Mycobacteria Research Laboratories, Department of Microbiology, Immunology and Pathology, Colorado State University, 1682 Campus Delivery, Fort Collins, Colorado, 80523, United States of America; 2 Foundation for Innovative New Diagnostics (FIND), Chemin des Mines 9, 1202, Genève, Switzerland; IPBS, FRANCE

## Abstract

Globally, tuberculosis is slowly declining each year and it is estimated that 37 million lives were saved between 2000 and 2013 through effective diagnosis and treatment. Currently, diagnosis relies on demonstration of the bacteria, *Mycobacterium tuberculosis* (*Mtb*), in clinical specimens by serial sputum microscopy, culture and molecular testing. Commercial immunoassay lateral flow kits developed to detect *Mtb* lipoglycan lipoarabinomannan (LAM) in urine as a marker of active TB exhibit poor sensitivity, especially in immunocompetent individuals, perhaps due to low abundance of the analyte. Our present study was designed to develop methods to validate the presence of LAM in a quantitative fashion in human urine samples obtained from culture-confirmed TB patients. Herein we describe, a consolidated approach for isolating LAM from the urine and quantifying D-arabinose as a proxy for LAM, using Gas Chromatography/Mass Spectrometry. 298 urine samples obtained from a repository were rigorously analyzed and shown to contain varying amounts of LAM-equivalent ranging between ~10–40 ng/mL. To further substantiate that D-arabinose detected in the samples originated from LAM, tuberculostearic acid, the unique 10-methyloctadecanoic acid present at the phosphatidylinositol end of LAM was also analyzed in a set of samples and found to be present confirming that the D-arabinose was indeed derived from LAM. Among the 144 samples from culture-negative TB suspects, 30 showed presence of D-arabinose suggesting another source of the analyte, such as disseminated TB or from non-tuberculosis mycobacterium. Our work validates that LAM is present in the urine samples of culture-positive patients in small but readily detectable amounts. The study further substantiates LAM in urine as a powerful biomarker for active tuberculosis.

## Introduction

Tuberculosis (TB) is a sub-acute or chronic infectious disease caused by *Mycobacterium tuberculosis* (*Mtb*), the bacillus that infects approximately one-third of the world’s population. According to the latest World Health Organization report, in 2013, an estimated 9.0 million people developed TB and 1.5 million died from the disease, 360,000 of whom were HIV co-infected. Fortunately, the global incidence of TB is slowly declining each year and it is estimated that 37 million lives were saved between 2000 and 2013 through effective diagnosis and treatment [1]. The report notes that “TB remains unique among the major infectious diseases in lacking accurate and rapid point-of-care tests, largely due to insufficient progress in biomarker discovery…the most pressing priority in TB diagnostics research today is the development of a simple, low-cost, instrument-free rapid test.”

Currently, sputum smear microscopy is the most widely used diagnostic method for TB. This method lacks adequate sensitivity and is time-consuming. Culture, which greatly improves sensitivity of detection, is slow and expensive and requires trained personnel. The traditional diagnostic approaches require the patient to make repeated trips to the clinic, which often is cost-prohibitive for the patient, resulting in the failed opportunity for early diagnosis and treatment (reviewed in [2]). In December 2010, WHO endorsed the Xpert MTB/RIF test for use in TB endemic countries [3] and declared it a major milestone for global TB diagnosis. Xpert MTB/RIF is a cartridge-based, automated diagnostic test that can identify *Mtb* DNA and mutations associated with resistance to rifampicin (RIF) by nucleic acid amplification technique (NAAT) [4–6]. A reliable biomarker, if detectable on a simple, portable, and low-cost platform such as currently deployed in much of the world for malaria and HIV, could facilitate early detection, reducing not only morbidity but also transmission, and supporting global TB control. Moreover, a specific biomarker that could reduce the size and duration of clinical trials for new drug candidates through better identification of treatment efficacy, disease activity, cure and relapse would have a huge impact on the cost of new drug development. Recently, biomarkers such as Interferon-γ-inducible protein 10 (IP-10) have been shown to be non-specific for TB [7] and transrenal DNA has been used for extrapulmonary-TB diagnosis [8–9]. Among the bacterial products, Lipoarabinomannan (LAM) has received intense attention in developing non sputum based diagnostic platforms. A commercially available urine LAM diagnostic test is available; however, poor sensitivity has led to limited use. [10–12]. Urinary LAM detection using a commercially available lateral flow immunoassay has been shown to have poor sensitivity, especially in patients without advanced HIV-related immunodeficiency and systemic tuberculosis in a number of studies [13]. In another approach, urinary LAM has been detected with 82% sensitivity and 100% specificity only after using a laborious magnetic nanoparticle based concentration step [14].

**Fig 1:**
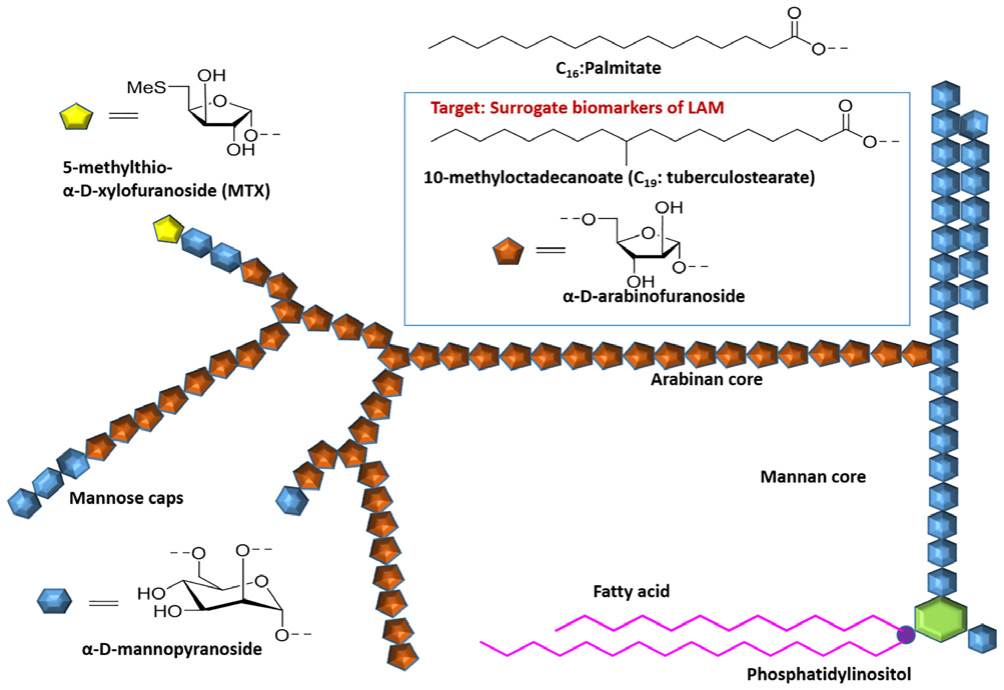
Representative schematic structure of ManLAM, Insert in the Blue box show residues adapted as strategic surrogates for LAM.

LAM is one of the three major groups of interrelated lipoglycans within the mycobacterial cell wall [15–17] which are non-covalently linked to the plasma membrane and or outermembrane via a phosphatidylinositol anchor and extend to the surface. LAM molecules have three major structural domains. The phosphatidylinositol anchor is linked to the mannan backbone which is, in turn, attached to a heterogeneous arabinan domain (Fig 1). Variable capping of the arabinan moiety with terminating mannose residues results in a diversity of LAM molecules in structure and functions [15, 18].

LAM with terminal mannose caps on the D-arabinan end (ManLAM) is characteristic of pathogenic, slow growing mycobacterial species such as *M*. *tuberculosis*, *M*. *leprae* and *M*.*bovis* [15–17]. The average molecular weight of LAM has been found to be approximately 17.3 kDa, with a broad distribution on either side that reflects considerable molecular heterogeneity with regard to size, pattern of branching of the arabinan side-chains, capping, acylation and branching of the mannan backbone [15–17].

There is some evidence suggesting that LAM is actively secreted from infected alveolar macrophages [19]. Such an active process would be consistent with the important immunomodulatory properties of LAM that are likely to favor survival of the organism *in vivo* [20]. This would result in LAM in the bloodstream which could pass into urine through glomerular filtration [21]. However, LAM is antigenic and thus may be bound in blood as immune complex, and less able to enter the urine in the setting of functioning glomerulus. Thus, the ratio between free LAM and immune-complexed LAM in bloodstream would be a critical factor in predicting the presence of free LAM in urine. A different mechanism for the presence of LAM in urine would be direct renal or urogenital tuberculous pathology resulting in mycobacteriuria [22,23]. Significantly, this mechanism does not require LAM, dissociated from *M*.*tuberculosis*, to pass through the kidney.

In order to validate the presence of LAM in the human clinical samples, we have developed a chemical approach utilizing specific derivatization and GC/MS analyses with the conviction that D-arabinose is only present in the mycobacteria and is absent among eukaryotes [24]. We hypothesized that D-arabinose, released from LAM by acid hydrolysis, will be a specific surrogate for LAM, unlike D-mannose which is abundant in the eukaryotes. However, even in the normal urine, substantial amounts of D-arabinose was found which led us to develop a method of purifying urinary LAM away from the endogenous glycan-containing arabinose. A method for the detection of low amounts of D-arabinose by GC/MS released from LAM by acid hydrolysis was developed using the tri-*O*-trifluoracetyl derivatives of (R)-2-octyl arabinosides. Using this method we analyzed 298 urine specimens provided by the Foundation for Innovative New Diagnostics (FIND) from well-characterized patients with or without culture-confirmed tuberculosis, all of whom presented with symptoms suggestive of active TB. D-arabinose derived from LAM was found to be present in varying amounts (~10–40 ng/mL) in the TB positive clinical samples.

In addition, the detection of tuberculostearic acid, the mycobacteria-specific 10-methyloctadecanoic acid by GC/MS, was also considered as a second option for unequivocal validation of LAM in urine. This unique fatty acid identified in the LAM class of molecules was analyzed as its pentafluorobenzyl ester by GC/negative ion chemical ionization mass spectrometry.

## Materials and Methods

### Materials

Most of the reagents were bought from Sigma-Aldrich and used without further purification. The silylation agent, HTP-trisil, was bought from Thermo-Fisher Scientific. Solvents were purchased from Fisher Scientific. Uniformly labeled ^13^C_5_-D-arabinose was purchased from Cambridge Isotope Laboratories, Inc. Octyl sepharose CL 4B was obtained from GE Healthcare. GC/MS was recorded on CP 3800 gas chromatograph (Varian) equipped with an MS320 mass spectrometer. Helium was used as the carrier gas with a flow rate of 1 ml/min. Argon was used as the collision gas in MS/MS experiments and methane was used as the chemical ionization gas for the negative chemical ionization analyses. The samples were analyzed on an Agilent J&W capillary column VF-5ms column (30 m × 0.25 mm i.d.). The data analyses were carried out on a Varian WS data station. NMR spectroscopy was carried out on Innova 400 (Varian) at the Central Instrument Facility, CSU. Healthy nonendemic urine (NEU) samples were collected from willing co-worker-volunteers in CSU. The primary antibody CS-35, used in the dot blot assay was developed in our laboratory and the detection antibody anti-mouse IgG alkaline phosphatase was purchased from Sigma-Aldrich

### Study Cohort

Anonymized archived urine samples used in our study were provided by the Foundation for Innovative New Diagnostic (FIND, Geneva). The study samples were collected from patients with symptoms of pulmonary tuberculosis presenting prior to the initiation of treatment to clinics in Vietnam, South Africa and Peru. All human urine specimens were collected from adult participants of both sexes suspected of pulmonary TB, with and without HIV co-infection. The authors are unware if the urine specimens were taken as a ‘second catch’ and ‘creatinuria’ was not used as internal control to account for the variations in the urine flow rate [25]. Urine specimens were sedimented by centrifugation and the supernatant was stored at -80°C within a few hours of collection. Final diagnosis (TB vs. non-TB) was established on the basis of microscopy plus >2 sputum cultures and clinical and radiologic examinations. TB was defined as being culture positive from at least one sample. Non-TB was defined as being smear and culture negative on all samples and having improved clinically/radiologically without TB-specific therapy. Patients without a firm final diagnosis (e.g. contaminated culture, persistent symptoms despite repeated negative TB cultures, or treatment for TB without culture-confirmation) were excluded from study.

### Ethics Statement

Anonymized archived urine samples used in our study were provided by the Foundation for Innovative New Diagnostic (FIND, Geneva) http://www.finddiagnostics.org/programs/tb/find_activities/tb_specimen_bank.html. The sample numbers presented here are arbitrary and cannot be traced back to individual patients.

The study was approved by the local IRB and all subjects providing samples signed an informed consent at the time of enrollment and before sample collection. Participants were informed that the samples were going to be stored at FIND repository and will only be used for the development of new TB diagnostics. None of the authors have access to identifying patient information.

### Sample Preparation and LAM Purification

All urine samples (3.5 mL each) were dialyzed (overnight) against deionized water with 3.5 kDa MWCO (Spectrumlab) membrane filter. The dialyzed urine was then dried and reconstituted in deionized water (500 μL). An aliquot (100 μL) of each sample was dried and re-suspended in 5% n-propanol in 0.1 M ammonium acetate (NH_4_OAc) (100 μL). This turbid solution was then loaded onto an OS-CL 4B column (750 μL OS suspension loaded in a 10 mL poly-prep chromatography column and centrifuged for 3 min at 3500 rpm; settled into 500 μL column volume; washed with 2 mL of 5% n-propanol in 0.1 M NH_4_OAc). Elution was started with 5% n-propanol in 0.1 M NH_4_OAc (4 mL), 15% n-propanol in 0.1 M NH_4_OAc (4 mL), 40% n-propanol in 0.1 M NH_4_OAc (2 mL) and 65% n-propanol in 0.1 M NH_4_OAc (2 mL). The 40% and 65% eluates were combined and analyzed.

#### Dot blot analysis of LAM

A nitrocellulose dot blot technique was used to monitor the presence of LAM in appropriate fractions eluted off an Octyl Sepharose column. Three aliquots (1 μl, 2 μl and 5 μl) of each fraction (40% & 65%) and H_37_Rv LAM (1 μg; as positive control) were spotted onto a nitrocellulose paper. It was then blocked with 2% bovine serum albumin (BSA) in Tris-Buffered Saline Tween 20 (TBST; pH-7.6) (10 mL) at 25°C for 45 min followed by overnight incubation at room temperature with 0.5 mL CS-35, anti-LAM monoclonal antibody in blocking solution (10 mL). After washing the paper with TBST (10 mL X 6), antibody bound to the LAM/paper was treated with alkaline phosphatase-conjugated anti-mouse IgG for 45 min. The membrane was then washed thoroughly with TBST (10 mL X 4) and exposed to NBT-BCIP substrate in the dark (10 min) to develop [26].

### Chemical Derivatization

#### Hydrolysis

Typically a sample aliquot (100 μL) containing D-UL-^13^C_5_- arabinose (200 ng), as internal standard, was taken in oven dried tubes (13X100) and treated with 200 mL of 2M aqueous trifluoroacetic acid (TFA) at 120°C for 2 h. The reaction tube was then cooled to room temperature and dried under nitrogen gas flow.

#### Octanolysis

The TFA hydrolysate was treated with R-(-)-2-octanol (100 μL) cooled (0°C) and TFA (20 μL; ~2 M) was then added to it. It was then heated at 120°C overnight (14 h). After cooling down to room temperature, it was dried. The residue thus obtained was subjected for appropriate derivatization.

#### Silylation

The dry octyl-arabinoside thus obtained was treated with Tri-Sil HTP reagent (100 μL), and heated at 80°C for 20 min. It was then cooled to room temperature and centrifuged. The supernatant was taken for GC/MS analysis.

#### TFAA esterification

Acetonitrile (CH_3_CN. 100 μL) was added to the dry octyl-arabinoside followed by the addition of TFA (10 μL) and then cooled to 0°C. It was treated with trifluoroacetic anhydride (TFAA) (200 μL) and heated at 55°C for 20 min. The sample was then cooled to room temperature and dried. The residue thus obtained was reconstituted in chloroform (CHCl_3_) (50 μL) and analyzed by GC/MS.

### General Protocol for Urinary Tuberculostearic acid (TBSA) Analysis

An aliquot of urine (700 μL) was extracted with CHCl_3_ (500 μLx2) and the organic layer was discarded. The aqueous layer was dried under reduced pressure and then re-suspended in aqueous ammonia (28% NH_4_OH: water = 1:3) (200 μL) and kept at room temperature with occasional shaking for 16 h. All the liquids were removed under N_2_-stream and 2.5% (v/v) H_2_SO_4_ (200 μL) was added to it (pH ~2). It was then extracted with CHCl_3_ (200 μL X2) and organic layers were collected together and dried. To neutralize the trace amount of acid, 28% NH_4_OH (100 μL) was added to it and dried again. The dry sample was then treated with acetonitrile (100 μL), diisopropyl ethylamine (50 μL) and 2,3,4,5,6-pentafluorobenzyl bromide (20 μL) at room temperature. After 20 min, the sample was dried and reconstituted in CHCl_3_ (100 μL) and analyzed by GC/MS.

### GC/MS analysis

For the 1-(α/β-*O*- (R)-2-octyl)- 2,3,5 tri-*O*- trifluoroactyl-D-arabinofurano/ pyranoside analysis, the oven temperature was held at 50°C for 1 min and programmed at 20°C/min to 150°C and then programmed at 2.5°C/min to 200°C. For the TMS-derivatives of the D-arabinose and the uniformly labeled ^13^C_5_- arabinose internal standard ions at *m/z* 204, 217 and 206, 220 respectively were monitored on a SIM scan program. The TFAA-derivatives were monitored by MS/MS using *m/z* 420.9 (parent ion) to 192.9 (daughter ion), and m/z 425.9 (parent ion) to 197.9 (daughter ion) respectively for D-arabinose and D-UL-^13^C_5_- arabinose as internal standard. For the TBSA analysis, the oven temperature was held at 50°C for 1 min and programmed at 30°C/min to 150°C and then 10°C/min to 300°C. The mass spectrometer was set for a Select Ion Monitoring (SIM) program in Chemical Ionization (CI) mode whereby the characteristic free fatty acyl anion at *m/z* 293.7 was monitored. GC/MS analyses were carried out using a CP 3800 gas chromatograph (Varian) equipped with an MS320 mass spectrometer.

## Result and Discussion

The aim of this work is to validate the presence of LAM and its quantification in clinical urine samples. We, therefore, adopted a chemical strategy of converting LAM into its constituent monosaccharides (i.e. D-arabinose, D-mannose.) using 2 M TFA hydrolysis, and then analyzing the liberated D-arabinose using GC/MS after proper chiral derivatization. We used the chiral alcohol, R-(-)-2-octanol, for glycosylation [27]. The hydroxyl groups were then trimethylsilylated for subsequent GC/MS analysis. Since D-Mannose is ubiquitous, we excluded it from our analysis.

### Optimization of Chemical Derivatization and GC/MS Protocol

**Fig 2:**
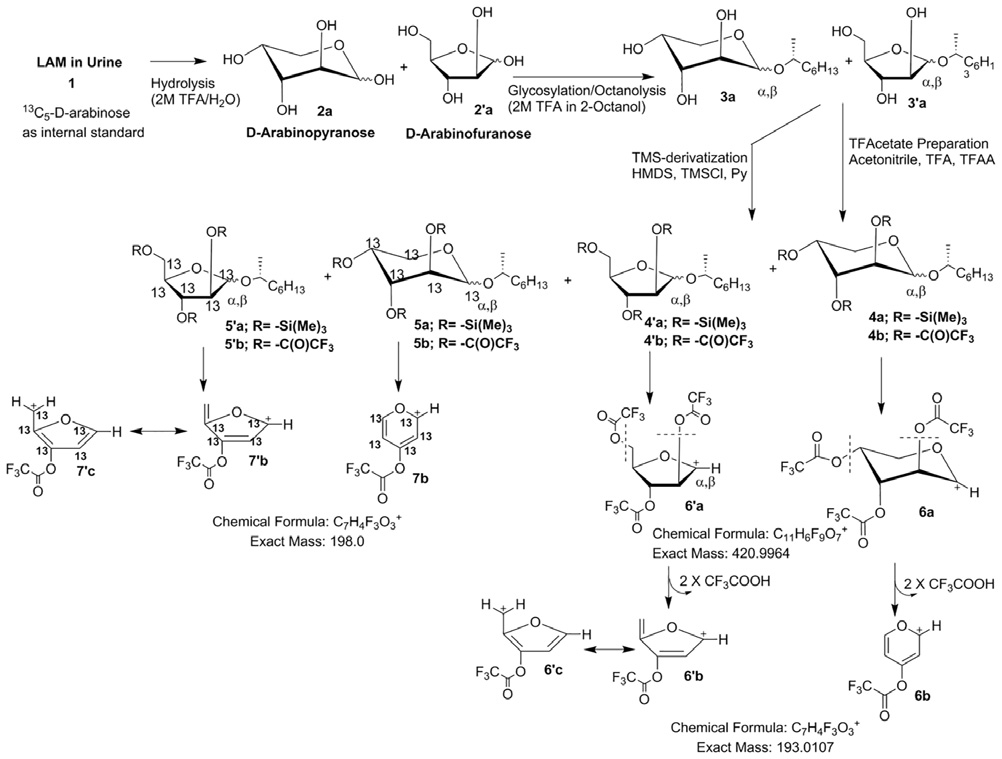
Protocol for derivatization of D-Arabinose in urinary LAM and the corresponding mass fragmentation pattern.

**Fig 3:**
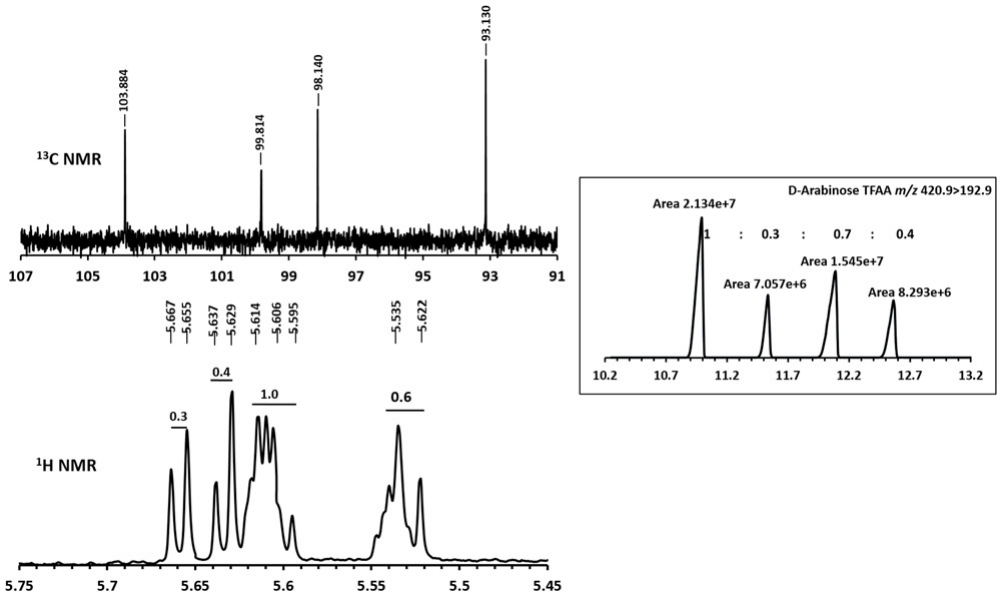
NMR spectra of anomeric protons and GC/MS peak ratio and different stereoisomeric forms of 2,3,5-trifluoroacetyl-1-(R-2-octyl)-arabinosyl glycosides.

**Fig 4:**
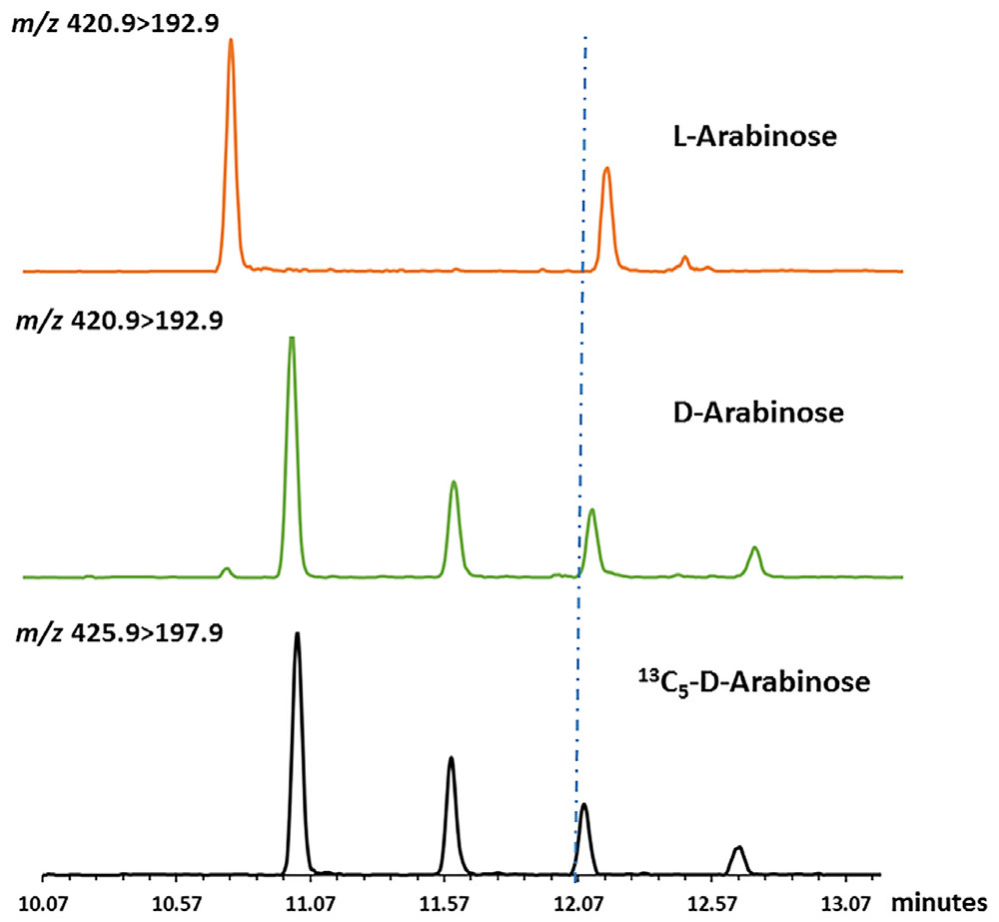
Differences in peak patterns and retention times in GC/MS chromatogram for D- and L- arabinose.

First we attempted to analyze urine samples from five TB sputum smear microscopy and culture positive (TBSSMC+) and five TB sputum smear microscopy and culture negative (TBSSMC-) urine samples. All the samples (3.5 mL each) were dialyzed, dried and reconstituted in 500 μL deionized water and an aliquot of 100 μL (i.e 0.7 mL of original urinary volume) was used for analysis. All the urine samples were also spiked with 200 ng of ^13^C_5_ D-arabinose as the internal standard (IS). After hydrolysis, octanolysis and TMS-derivatization (Fig 2; **4a, 4′a**) the samples were subjected to GC/MS analysis. Unfortunately, the four expected diagnostic peaks (α,β mixtures of furanose, and pyranose) for D-arabinose overlapped with contaminant peaks thereby compromising the sensitivity and accuracy in quantitation. Therefore, we opted for an alternative of trifluoroacetate as oppose to TMS derivatives [28]. Derivatization of the 1-*O*-(R)-2-octyl-D-arabinosides (Fig 2; **3a, 3′a**) was carried out with trifluoroacetic anhydride to make the mixture of 1-(α/β-*O*-(R)-2-octyl)- 2,3,5 tri-*O*- trifluoroactyl-D-arabinofurano/pyranosides (Fig 2; **4b, 4′b**). These derivatives produced four diagnostic peaks at *m/z* 420.9 (Fig 2; **6a, 6′a**) *via* loss of *O*-octyl aglycon in GC/MS. All four stereoisomers resolved well on the GC with specific retention times. The ion (*m/z* 420.9; **6a, 6′a**) underwent further fragmentation in an MS/MS experiment giving a daughter ion corresponding to the loss of two trifluoroacetic acid residues at *m/z* 192.9 (**6b, 6′b–c**).

We envisaged that the detection of D-arabinose by monitoring the conversion of *m/z* 420.9 (**6a, 6′a**) to *m/z* 192.9 (**6b, 6′b–c**) was attributable to pentoses only and D-arabinose was confirmed by its retention time compared to the retention time of the IS for which ions were monitored at *m/z* 425.9 (**7a, 7′a**) and *m/z* 197.9 (**7b, 7′b–c;** Fig 2).

Importantly, since all five C-atoms of arabinose are retained in the corresponding fragment ions, the mass difference between D-arabinose and that of IS (^13^C_5_-D-arabinose) remains 5 amu. The fact that the total amount of D-arabinose in any given sample is distributed over four-peaks can be attributed to the presence of different stereoisomers of D-arabinose–derivative arising from the furanose and pyranose conformations and their anomeric mixtures (α and β) formed due to the non-stereocontrolled glycosylation conditions used. The ^13^C-NMR of 1-(α/β-*O*- (R)-2-octyl)- 2,3,5 tri-*O*- trifluoroactyl-D-arabinofurano/pyranoside (**4b, 4′b**) revealed the presence of four anomeric carbons at δ ppm 103.8, δ ppm 99.8, δ ppm 98.1 and δ ppm 93.1 which can be attributed to α/β-furanoside/pyranoside conformations of D-arabinose derivative. Importantly, the integral values of the anomeric protons at δ ppm 5.65 (d), δ ppm 5.63 (d), δ ppm 5.60 (m) and δ ppm 5.53 (m) in the ^1^H NMR spectrum of **4b** and **4′b** bear a comparable ratio to the peak intensities obtained from GC/MS thereby supporting our contention that the chiral glycosylation of D-arabinose produces 4-peaks in a GC/MS analysis, on achiral GC-column, arising from the relative abundance of different anomers-conformers. (Fig 3; also see S1 Fig).

At this point, it was necessary to verify if L-arabinose and D-arabinose can be differentiated by this protocol. L-Arabinose (200 ng) along with the IS (200 ng) was also subjected to derivatization and analysis by GC/MS. The difference in peak patterns and retention time(s) (Fig 4) between D- and L-arabinose were evident in the chromatogram.

### Optimization of Urine LAM Analysis

Equipped with better derivatization protocol and cleaner GC/MS method, ten clinical (five TBSSMC + and five TBSSMC–) samples and one healthy nonendemic urine (NEU) sample as a negative control were analyzed. Unfortunately, all eleven samples, irrespective of clinical status were found to contain D-arabinose. The NEU sample also showing the presence of D-arabinose pointed towards the presence of D-arabinose containing polymer(s) or polysaccharide(s) contaminants. No free D-arabinose was expected to be present as the samples were pre-dialyzed with a 3.5 kDa cut off membrane.

We suspected that D-arabinose, a rare pentose in eukaryotes, is found in urine as neutral polysaccharide(s) such as hemicellulose, pectins and/or arabinoxylans originating from everyday food and vegetables [29]. Therefore, we directed our efforts towards the pretreatment of urine prior to chemical derivatization and GC/MS analysis. Importantly, LAM being the only D-arabinose-containing lipoglycan known so far, we wanted to separate out LAM from endogenous D-arabinose containing polysaccharide contaminant(s) by way of hydrophobic interaction chromatography (HIC). Owing to the amphipathic nature of LAM, it has been shown [30, 31] to be able to be resolved from neutral glycans on an octyl-sepharose Cl-4B (OS) using 5–65% n-propanol in 0.1 M ammonium acetate (NH_4_OAc) solvent gradients. Typically, neutral components that do not bind to OS are eluted in the 5–15% n-propanol in 0.1 M NH_4_OAc fractions whereas LAM, as it binds to OS, is eluted using 40–65% n-propanol in 0.1 M NH_4_OAc. In a control experiment, two portions (each 0.5 mL) of a healthy NEU sample were spiked with 1 μg and 0.5 μg of LAM respectively and subjected to HIC purification using stepwise solvent gradient starting from 5%, 15%, 40% and 65% n-propanol in 0.1 M NH_4_OAc. In dot-blot assay, as described in the experimental section, all 40% eluates were found to be LAM-positive and the 65% fractions were found to contain no LAM. While most of the spiked LAM eluted in the 40%-fraction, we could not rule out the possibility that the 65% fractions may also contain trace amount of LAM which is probably below the detection limit of a dot blot.

**Fig 5:**
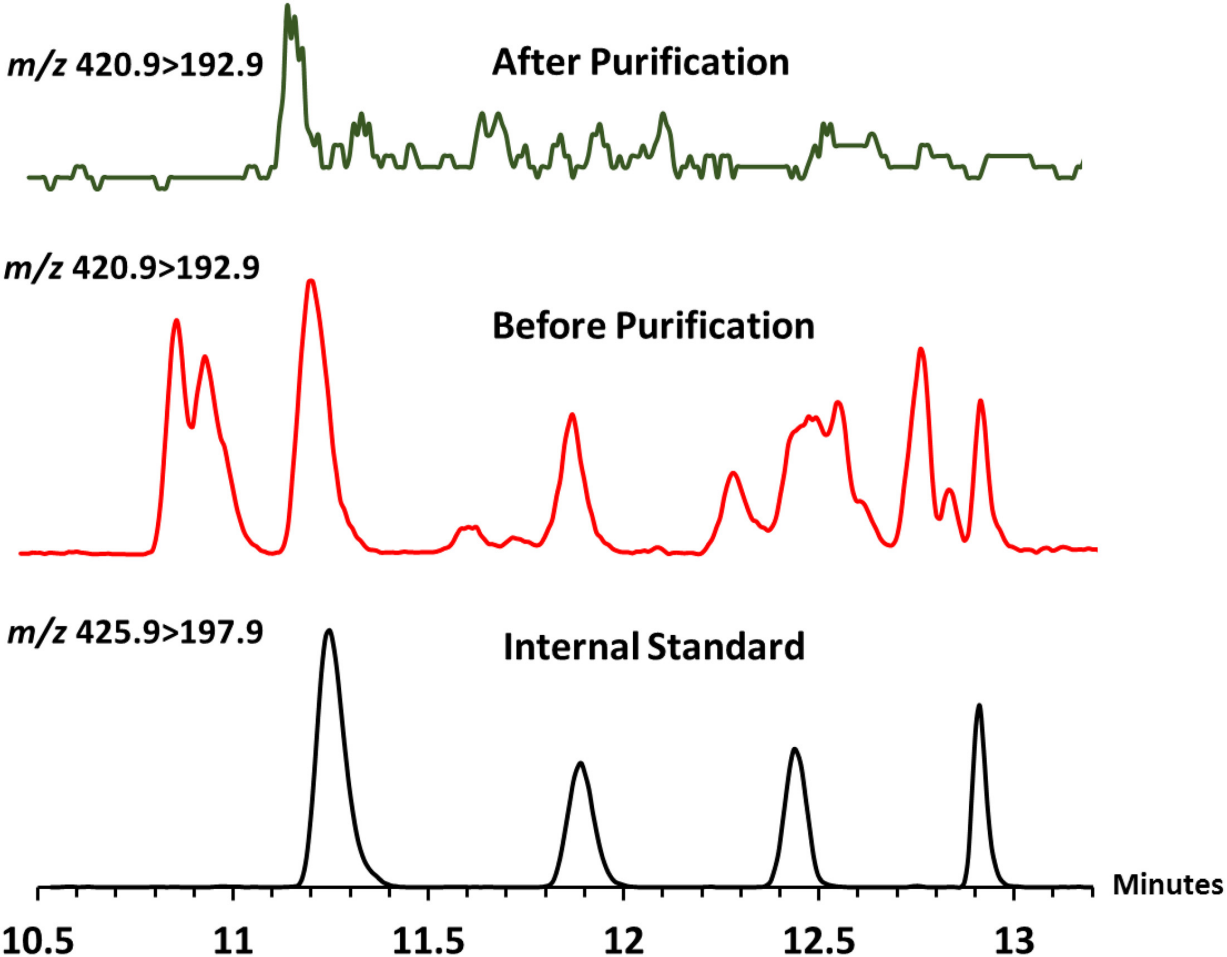
D-Arabinose estimation of Non-Endemic-Urine (NEU). 1) Internal standard, 2) D-arabinose in NEU before purification; 3) D-arabinose not detected in NEU after purification.

At this point, it was necessary to verify if HIC is efficient enough to remove D-arabinose containing polysaccharide contaminants present in NEU. Two aliquots (1 mL each) of NEU were taken; one aliquot was passed through the OS-column and another was not. To our satisfaction, the OS-column passaged NEU revealed no detectable D-arabinose when analyzed by GC/MS. On the contrary, the NEU aliquot not passed through the OS-purification revealed the presence of considerable amount of D-arabinose (Fig 5). However, quantification was needed in order to monitor the recovery and determine the limit of detection (LOD). For this, four aliquots (1.0 mL each) of dialyzed NEU were spiked with 500 ng (A), 50 ng (B), 5 ng (C) and 0.5 ng (D) of LAM respectively. All the NEU samples were then subjected to OS-column followed by addition of IS (200 ng), derivatization and GC/MS analysis (see S2 Fig). It was envisaged that A would reveal the recovery efficiency of this entire protocol while B, C and D might give us the LOD. A recovery of 360 ng (72%) of LAM (details of calculation is mentioned later in this section) was revealed from A. However, we were able to see all four peaks of D-arabinose in B and C distinctly but not in D. Therefore, we found our LOD around 5 ng without taking into account the loss of sample during purification and incomplete octanolysis as discussed below.

With the development of an efficient urinary LAM purification protocol and sensitive method of analysis we set out to analyze the clinical samples. Cautiously, we took 100 μL aliquots (amounts to 0.7 mL i.e., 1/5^th^ of 3.5 mL of original urine) of dialyzed, dried and reconstituted urine samples of which two were TBSSMC+ (**K49** and **K 70**; Table 1) and two were TBSSMC- samples (**K174** and **K144**; Table 1). These samples were subjected to the purification protocol and 40–65% octyl sepharose eluents were combined together. After usual processing and derivatization they were analyzed using GC/MS. The TBSSMC+ samples had measurable amounts of D-arabinose whereas D-arabinose was not detected in these two TBSSMC- samples.

**Table 1:** FIND urine sample purification and D-arabinose analysis. IS = Internal Standard; HIV = Human Immunodeficiency Virus; Pneu = Pneumonia; Atyp = Atypical TB,

TBSSMC + Urine samples													
Sample ID	Other infec.	1^st^ run										2^nd^ run	
		(*p*_1_)	(*p*_2_)	(*p*_3_)	(*p*_4_)	(*I*_*1*_)	(*I*_*2*_)	(*I*_*3*_)	(*I*_*4*_)	(ng) (*A*_0_)^*a*^	ng/mL (*L*_1_)	(ng) (*A*_0_)	ng/mL (*L*_1_)
**K49**		**19**	**10**	**143**	**19**	**350**	**141**	**2214**	**454**	**10.8**	**25.8**	**19.0** ^b^	**22.5**
**K70**		**43**	**16**	**168**	**11**	**937**	**574**	**4168**	**682**	**6.5**	**15.3**	**14.0** ^b^	**16.6**
**K151**		**25**	**20**	**101**	**7**	**897**	**453**	**3753**	**713**	**5.5**	**13.2**	**14.6** ^b^	**17.3**
**K201**	**HIV**	**1240**	**432**	**652**	**273**	**27853**	**10273**	**17120**	**6347**	**8.9**	**21.1**	**9.2** ^a^	**21.9**
**K202**	**HIV**	**46**	**39**	**354**	**40**	**582**	**302**	**3550**	**412**	**15.8**	**37.5**	**34.5** ^b^	**41.0**
**K203**	**HIV**	**3601**	**1810**	**1043**	**659**	**82445**	**40998**	**34093**	**15684**	**7.5**	**17.8**	**15.0** ^b^	**17.8**
**K204**	**HIV**	**2740**	**1005**	**675**	**402**	**93159**	**37044**	**18412**	**12983**	**5.8**	**14.0**	**20.0** ^b^	**23.8**
**K205**	**HIV**	**2598**	**909**	**921**	**367**	**75236**	**28233**	**15534**	**9419**	**6.9**	**16.4**	**7.5** ^a^	**17.8**
**K206**	**HIV**	**287**	**127**	**438**	**179**	**12088**	**5212**	**20112**	**7408**	**4.7**	**11.3**	**6.2** ^a^	**14.7**
**K207**	**HIV**	**67**	**28**	**110**	**33**	**1677**	**901**	**4651**	**916**	**7.9**	**19.0**	**5.1** ^a^	**12.2**
**TBSSMC—Urine samples**													
**K208**		**0**	**0**	**0**	**0**	**519**	**343**	**3291**	**691**	**0.0**	**0.0**	**0.0** ^c^	**0.0**
**K196**	**HIV/Pneu**	**0**	**0**	**0**	**0**	**263**	**184**	**1739**	**345**	**0.0**	**0.0**	**0.0** ^c^	**0.0**
**K184**	**Pneu/Atyp**	**14**	**0**	**107**	**11**	**292**	**189**	**1678**	**182**	**8.0**	**19.0**	**12.8** ^c^	**18.2**
**K174**		**0**	**0**	**0**	**0**	**200**	**163**	**1199**	**134**	**0.0**	**0.0**	**0.0** ^c^	**0.0**
**K144**		**0**	**0**	**0**	**0**	**388**	**222**	**1568**	**207**	**0.0**	**0.0**	**0.0** ^c^	**0.0**
**K111**		**0**	**0**	**0**	**0**	**1811**	**1303**	**9178**	**920**	**0.0**	**0.0**	**0.0** ^c^	**0.0**
**K83**		**0**	**0**	**0**	**0**	**4803**	**2668**	**15376**	**2797**	**0.0**	**0.0**	**0.0** ^c^	**0.0**
**K74**		**0**	**0**	**0**	**0**	**2931**	**1908**	**9381**	**1678**	**0.0**	**0.0**	**0.0** ^c^	**0.0**
**K17**		**0**	**0**	**0**	**0**	**3602**	**2773**	**11574**	**1661**	**0.0**	**0.0**	**0.0** ^c^	**0.0**
**K81**	**HIV**	**0**	**0**	**0**	**0**	**551**	**498**	**3300**	**260**	**0.0**	**0.0**	**0.0** ^c^	**0.0**

^*a*^ 1/5 of 3.5 mL sample analyzed,

^*b*^ 2/5 of 3.5 mL sample analyzed,

^c^ 1/3 of 3.5 mL sample analyzed

### LAM Quantitation

For quantitation, theoretically the ratio of internal standard to sample arabinose can be used for any one of the four isomeric peaks of the ^13^C_5_-D-arabinose/^12^C_5_-D-arabinose since both the stable isotope labeled internal standard and the unlabeled D-arabinose in the sample will give identical ratios of the α and β anomers of the pyranosyl and furanosyl ring forms for a given sample (the ratio may vary between samples). As it turned out, that all four peaks were clean of contaminants as shown by a consistency of the ^12^C-D-arabinose/^13^C-D-arabinose and therefore all four peaks were used in the calculation. This lead to Eq 1 to determine the amount of arabinose in the tube:

$$A_{0}=\;\frac{1}{4}\left(\frac{p_{1}}{I_{1}}+\frac{p_{2}}{I_{2}}+\frac{p_{3}}{I_{3}}+\frac{p_{4}}{I_{4}}\right)X\;200\;ng$$ (1)

Where A_0_ is the amount of arabinose in the tube in ng, (*p*_*1*_, *p*_*2*_, *p*_*3*_, *p*_*4*_) are the areas of the ion from ^12^C_5_-D-arabinose and (*I*_*1*_, *I*_*2*_, *I*_*3*_, *I*_*4*_) are the areas of the ion from the internal standard ^13^C_5_-D-arabinose.

The amount of LAM per milliliter of was then calculated by Eq 2:

$$L_{1}=\;\frac{A_{0}}{0.6}/\left(n\right)\;ng/mL$$ (2)

Whereas the division of *A*_*0*_ by 0.6 provided us the estimated amount of LAM analyzed since D-arabinose content in LAM is approximately 60% of its molar mass based on literature [15, 32]. This LAM-equivalent amount was further divided by the volume of the sample used for the analysis (n) to determine the amount of LAM present per mL of urine (*L*_*1*_).

### Analysis of Clinical Samples

In order to verify the reproducibility, the same four clinical samples were subjected to repeat purification, derivatization and GC/MS analysis. The results were consistent with the previous run as the amount of detected D-arabinose was proportional to the volume of urine i.e., same amount of LAM is present when calculated per mL of urine (deviation range 8–12%). Thus, two different runs for the same sample(s) produced the same results and more importantly, D-arabinose was not detected within the limits of detection in the clinically assigned TBSSMC- samples.

We next directed our effort to purify and analyze eight more TBSSMC+ urine samples (Table 1) and eight more TBSSMC- urine samples (Table 1). The samples **K49**, **K70** and **K151** were TBSSMC+ with no other reported co-infection whereas for samples **K201-K207** subjects were reported to be TBSSMC+ with HIV co-infection. Specifically for the samples **K196** and **K81** were reported to be with HIV infection and **K184** was suspected with either Pneumonia or Atypical TB infection. The results are summarized in Table 1. In our analysis, all TBSSMC+ samples had varying amounts of D-arabinose. On the contrary, for the TBSSMC- samples, except sample **K184**, D-arabinose (chromatograms presented in S3 Fig) could not be detected. This could be due to either the absence of LAM in the sample or an amount of D-arabinose below the limit of detection. A repeat analysis of the same samples yielded similar quantities of D-arabinose confirming (Table 1) the validity of the method.

Although the detected amount of D-arabinose was remarkably reproducible when the same sample was analyzed second time as shown in Table 1, the area of the *m/z* 197.9 peak produced by the internal standard for each of the four isomers varied considerably. The extreme example of this can be noted by comparing the analysis of line 6 versus line 1 of Table 1. Since the arabinose from the internal standard and the sample differ only by the isotope of the carbon atoms, this issue does not compromise the final calculation of arabinose in the sample as both labeled and unlabeled arabinose behave identically [33]. However, the ultimate sensitivity of the method is dependent on the recovery of clean D-arabinose. We believe this problem occurs due to the presence of alcohols, including the n-propanol used to elute the LAM from the OS column, which may interfere or compete with the octanolysis. We are continuing our efforts to overcome this problem but clearly the method is adequately robust and reproducible in its present form to detect LAM in the urine of TBSSMC+ cases.

To confirm that the D-arabinose was a valid surrogate marker of LAM, analysis of additional one hundred seventy eight of the urine samples was carried out. Among these, ninety three were from TBSSMC+ patients and eighty five were from TBSSMC- patients (S1 Table). Significantly, the analysis (some samples tested twice) revealed that all but seven of the ninety three TBSSMC+ patient samples have D-arabinose in concentrations ranging from 4.4 to 42.8 ng/mL. On the other hand, there was no quantifiable D-arabinose or LAM in seventy four of the eighty five (87%) TBSSMC- (S1 Table) patients.

We then analyzed a blinded cohort of 100 urine samples, i.e. without any clinical information prior to analysis. Results are summarized in S1 Table. These 100 samples were unblinded and clinical information obtained only after GC/MS analyses were completed. Remarkably, 49 of 51 urine samples from TBSSMC+ patients (98%), were found to contain LAM in our analysis. In this blinded cohort, eighteen, out of the forty-nine TBSSMC- patients (37%), appeared to contain LAM. There could be two possibilities for these results. The first is that these patients may in fact have had TB that has not been diagnosed by serial microscopy and culture at presentation and follow-up. Given that the overall prevalence of tuberculosis in these cohorts was less than 37%, prior to microbiologic evaluation, it is highly unlikely that a third of culture-negative patients had tuberculosis. The second is that the D-arabinose comes from another source, such as infection or colonization with non-tuberculosis mycobacteria (NTM). At present, we cannot distinguish between the two possibilities although ultimate methods of LAM-based diagnostics in urine might be able to do so. Also, even if NTM infection is the source of the apparent LAM in these specimens, it is a small enough portion of TB negative patients that the presence of LAM in urine still correlates strongly, if not perfectly, with tuberculosis active disease.

### Analysis of Urinary Tuberculostearic Acid

At this point, we sought confirmation of the presence or absence of LAM by using a different surrogate marker than D-arabinose. We therefore, opted for the detection of the urinary tuberculostearic acid (TBSA), a 10-methyloctadecanoic acid and unique component of LAM with 1–2% molar mass distribution [15, 32]. We argued that if both D-arabinose and TBSA can be detected in the same clinical sample, they should originate from LAM.

**Fig 6:**
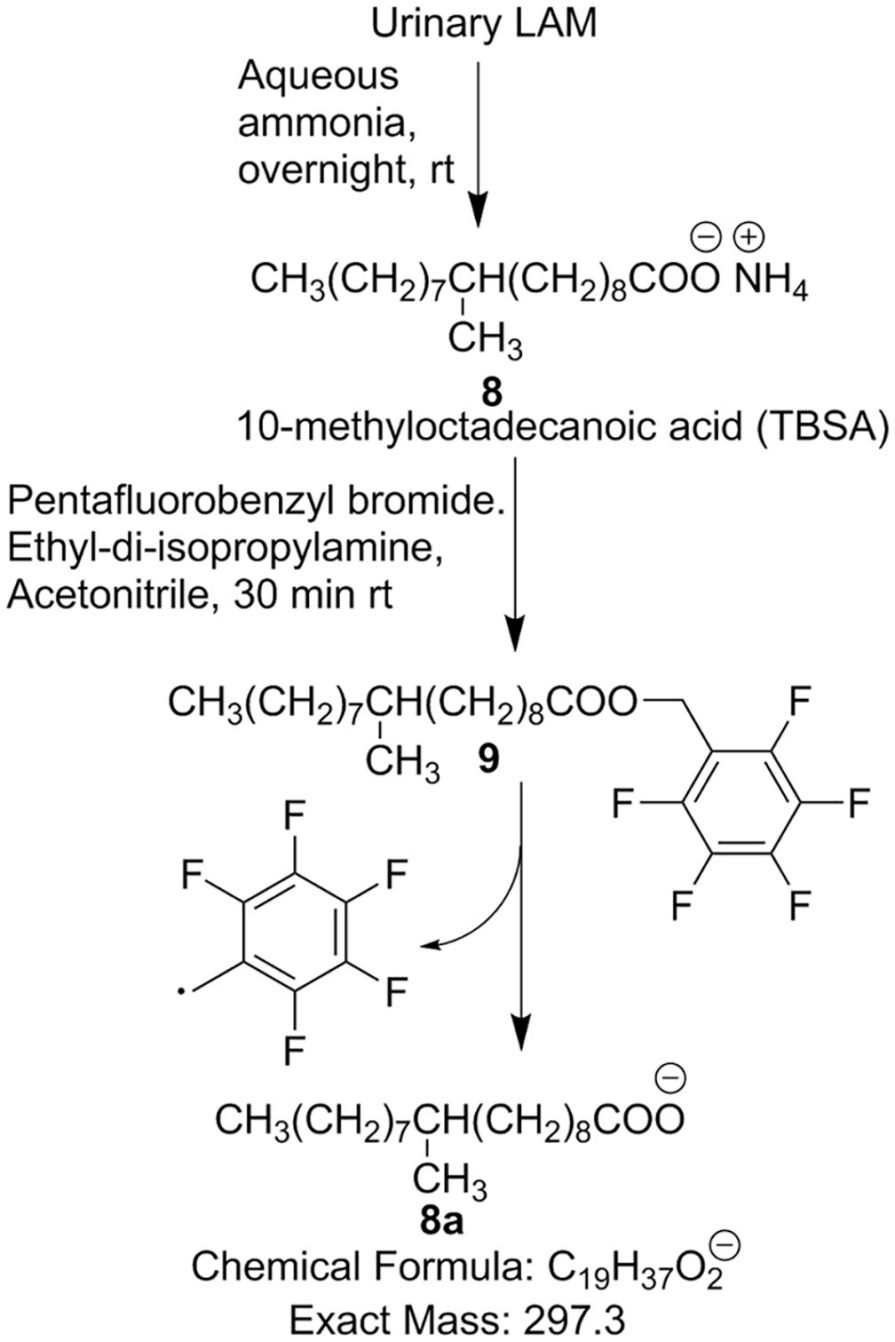
Protocol for TBSA detection by GC/MS.

For the detection of TBSA by GC/MS, we followed the procedure whereby a pentafluorobenzyl ester derivative of TBSA derived from sputum was made and analyzed by GC/MS using chemical ionization (CI) in negative mode detecting the characteristic mass fragment *m/z* 297.3 [34]. We modified the procedure by extraction with chloroform instead of hexane and hydrolysis with aqueous ammonia, a volatile base, instead of sodium hydroxide Fig 6.

**Fig 7:**
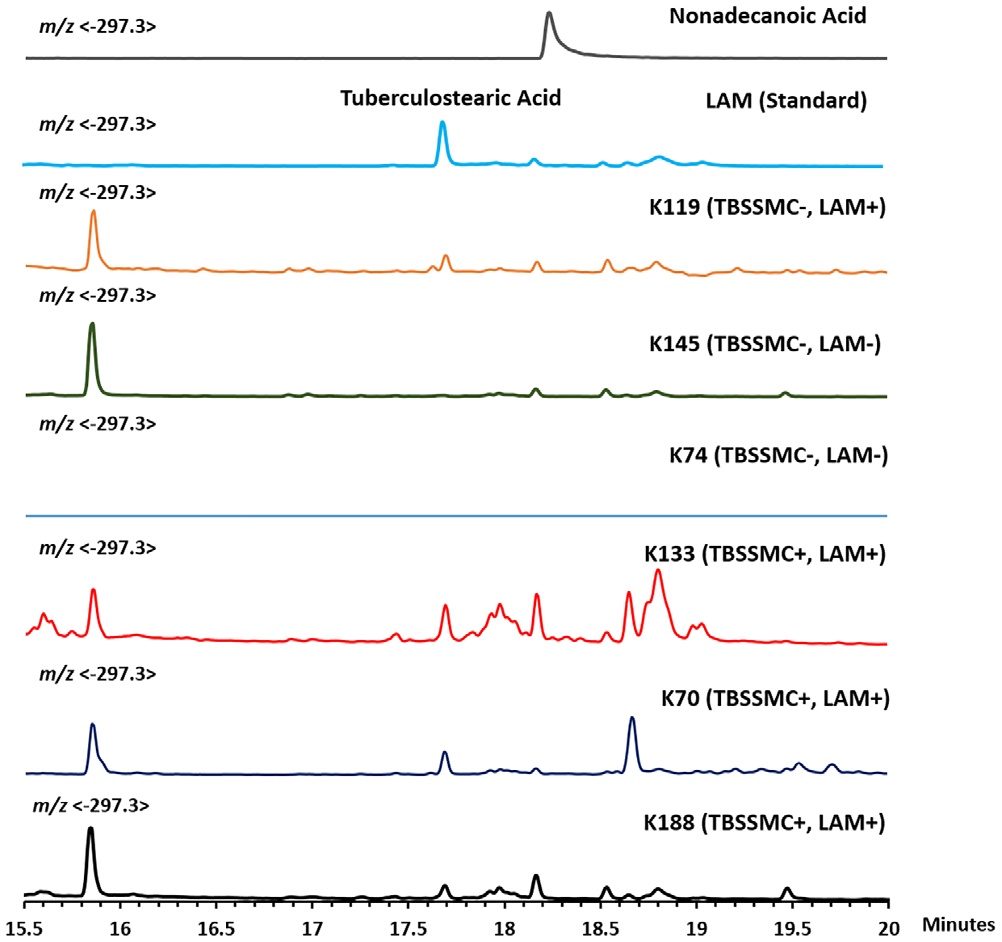
GC/MS chromatogram for TBSA detection in TBSSMC+ and TBSSMC- urine samples.

We analyzed six representative urine samples among which three were TBSSMC+ and three were TBSSMC-, including **K119**, a TBSSMC- urine sample previously found to have detectable amount of D-arabinose. We also included LAM and nonadecanoic acid as controls. As expected, all the three TBSSMC+ samples as well as **K119** revealed the presence of TBSA while the rest of the samples were devoid of TBSA (Fig 7).

Further, we subjected twenty eight (as available) TBSSMC- but D-arabinose positive urine samples for TBSA analysis. All samples had detectable amount of TBSA. A GC/MS experiment was done using known amount of LAM and the amount of TBSA was calculated based on D_2_-palmitic acid (used as IS; 20 ng). This experiment revealed that the approximate TBSA content in LAM is about ~0.9%. Based on this, the amount of LAM-equivalent determined for the TBSA-positive samples matched closely with LAM equivalent based on D-Arabinose (ng/mL) (Table 2). We analyzed all the eight TBSSMC+ samples which lack the presence of measurable D-arabinose in our assay. Significantly, none of the samples revealed any detectable TBSA (S4 Fig). These results support the contention that D-arabinose can be detected and estimated as surrogate biomarker of urinary LAM along with TBSA detection.

**Table 2:** TBSA Based LAM Estimation of 29 Urine Samples (TBSSMC -, D-Arabinose/LAM positive).

Samples	TBSA/LAM-eq (ng/mL)	D-Ara/LAM-eq (ng/mL)	Samples	TBSA/LAM-eq (ng/mL)	D-Ara/LAM-eq (ng/mL)
**B115**	22.5	23.3	**B6**	39.4	29.7
**B116**	27.5	34.6	**B64**	38.8	39.2
**B12**	30.6	21.4	**B79**	21.6	18.5
**B123**	41.9	47.6	**K108**	22.2	7.8
**B125**	27.4	30	**K116**	27.4	20.2
**B134**	21.3	25	**K119**	10.3	12.6
**B14**	30.4	26.6	**K161**	34.5	31
**B141**	47.9	54.1	**K181**	27.2	14.3
**B15**	34.0	32.1	**K184**	23.4	19
**B150**	44.6	48.7	**K40**	22.3	14.3
**B2**	12.3	13.5	**K5**	21.3	18.5
**B23**	17.1	29.7	**K60**	18.2	10.8
**B24**	26.3	16.6	**K89**	34.5	31.4
**B3**	15.7	19.6	**K93**	19.8	16.7
**B5**	35.1	19			

## Conclusion

**Fig 8:**
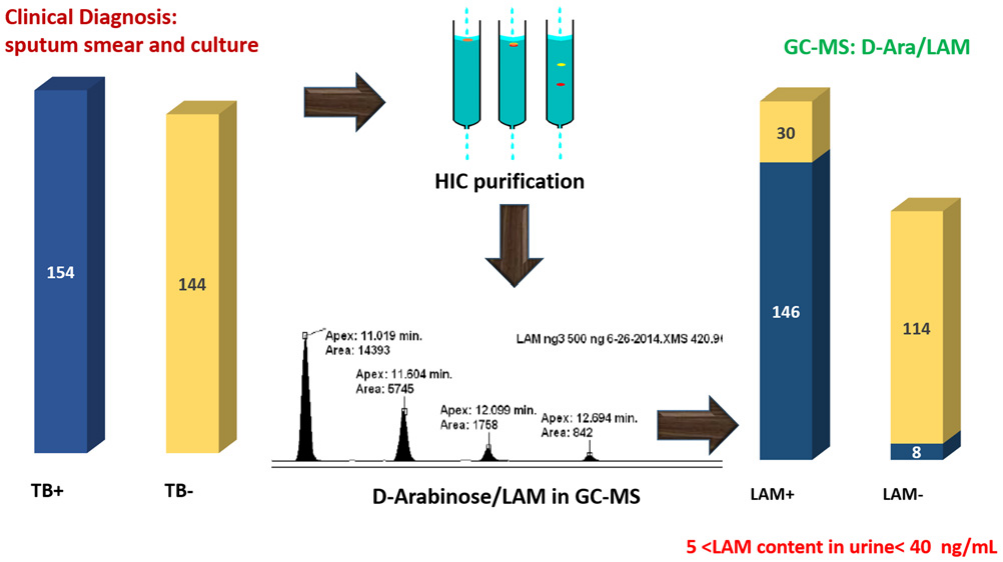
Summary of results for the GC/MS based urinary LAM detection on clinical samples.

We have been able to detect and measure urinary LAM in human clinical specimens by quantification of D-arabinose and TBSA using chemical derivatization and GC/MS. Our report includes analyses of over 298 urine samples, of which 100 samples were analyzed blinded. We established that normal and infected urine samples have significant amounts of non-dialyzable endogenous D-arabinose that needs to be removed prior to the analysis of LAM. For this, urine samples were passed through hydrophobic interaction chromatography to eliminate endogenous D-arabinose containing polymer. LAM was found to be present in varying amounts ranging between 5-40ng/mL of urine (Fig 8). Importantly, this chemical approach of detecting urinary LAM using GC/MS is non-discriminatory, i.e. this method does not depend on country of origin, HIV co-infection or BCG-vaccination. The origin of urinary LAM in ~20% of the culture negative urine specimens is unclear and needs to be explored further.

## Supporting Information

S1 Method Method Protocol for Dot blot immunoassay. (DOCX)

S1 Fig ^1^H NMR spectrum and ^13^C NMR spectrum of 2,3,5-trifluoroacetyl-1-R-2-octyl-D-arabinosides. (DOCX)

S2 Fig Recovery from HIC and Limit of detection for LAM determined by GC/MS analysis of D-arabinose. (DOCX)

S3 Fig Representative GC/MS Chromatograms for TBSSMC+ and TBSSMC- urine samples. (DOCX)

S4 Fig TBSA analysis of 8 urine samples which were TBSSMC + but was found to be LAM negative by GC/MS. (DOCX)

S1 Table FIND urine sample and 100 Blind FIND urine samples: D-Ara analysis. (DOCX)
