# Supplementary material for: Estimation of D-Arabinose by Gas Chromatography/Mass Spectrometry as Surrogate for Mycobacterial Lipoarabinomannan in Human Urine
Source: PLoS One. 2015 Dec 3;10(12):e0144088. doi: 10.1371/journal.pone.0144088 (PMC4669150; doi:10.1371/journal.pone.0144088)
Supplement: S1 Method — (DOCX) [file pone.0144088.s001.docx]

**S1 Method. Dot blot analysis of LAM.** A nitrocellulose dot blot technique was used to monitor the presence of LAM in appropriate fractions eluted off an Octyl Sepharose column. Three aliquots (1 μl, 2 μl and 5 μl) of each fraction (40% & 65%) and H_37_Rv LAM (1 μg; as positive control) were spotted onto a nitrocellulose paper. It was then blocked with 2% bovine Serum Albumin (BSA) in Tris-Buffered Saline Tween 20 (TBST; pH-7.6) (10 mL) at 25°C for 45 min followed by overnight incubation at room temperature with 0.5 mL CS-35, anti-LAM monoclonal antibody in blocking solution (10 mL). After washing the paper with TBST (10 mL X 6), antibody bound to the LAM/paper was treated with alkaline phosphatase-conjugated anti-mouse IgG for 45 min. The membrane was then washed thoroughly with TBST (10 mL X 4) and exposed to NBT/BCIP substrate in the dark (10 min) to develop. ^1^

^1^Sharma, A., Saha, A., Bhatterjee, S., Majumdar, S. and Das Gupta, S.K. *Clinic. Vaccine Immunol.* **2006**, *13*, 1143-1154.
