# Supplementary material for: Estimation of D-Arabinose by Gas Chromatography/Mass Spectrometry as Surrogate for Mycobacterial Lipoarabinomannan in Human Urine
Source: PLoS One. 2015 Dec 3;10(12):e0144088. doi: 10.1371/journal.pone.0144088 (PMC4669150; doi:10.1371/journal.pone.0144088)
Supplement: S1 Fig — (DOCX) [file pone.0144088.s002.docx]

^1^H NMR

**Fig S-1: ^1^H NMR spectrum of 2,3,5-trifluoroacetyl-1-R-2-octyl-D-arabinosides**

^13^ C NMR

**Fig S-1: ^13^C NMR spectrum of 2,3,5-trifluoroacetyl-1-R-2-octyl-D-arabinosides**
