# Supplementary material for: Estimation of D-Arabinose by Gas Chromatography/Mass Spectrometry as Surrogate for Mycobacterial Lipoarabinomannan in Human Urine
Source: PLoS One. 2015 Dec 3;10(12):e0144088. doi: 10.1371/journal.pone.0144088 (PMC4669150; doi:10.1371/journal.pone.0144088)
Supplement: S3 Fig — (DOCX) [file pone.0144088.s004.docx]

**Fig. S-3: Representative GC/MS Chromatograms for TBSSMC+ and TBSSMC- urine samples**
