# Supplementary figures and images for: Estimation of D-Arabinose by Gas Chromatography/Mass Spectrometry as Surrogate for Mycobacterial Lipoarabinomannan in Human Urine
Source: PLoS One. 2015 Dec 3;10(12):e0144088. doi: 10.1371/journal.pone.0144088 (PMC4669150; doi:10.1371/journal.pone.0144088)

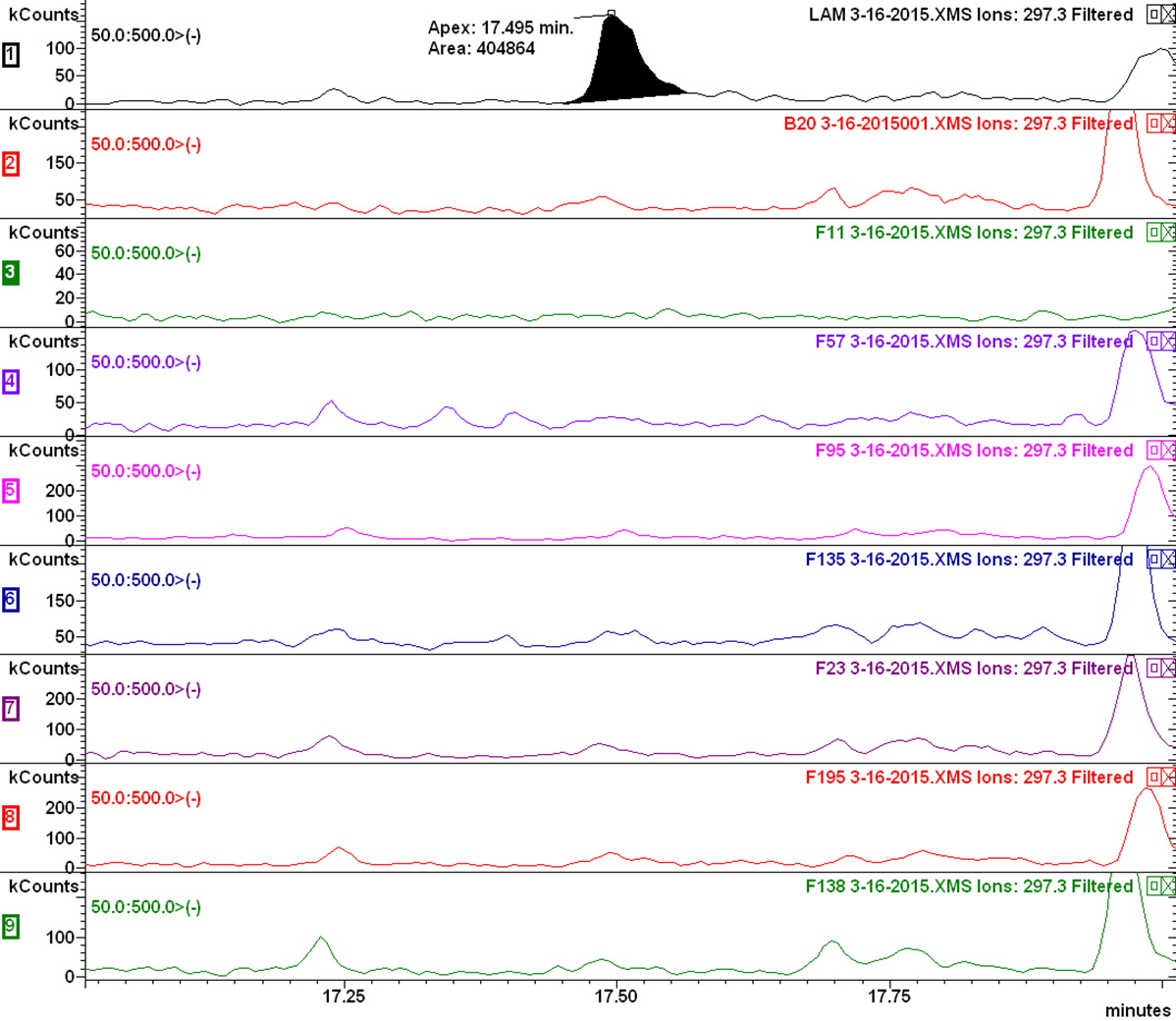


**Fig. S-4: TBSA analysis of 8 urine samples which were TBSSMC + but was found to be LAM negative by GC/MS**

Supplement: S4 Fig — (DOCX) [file pone.0144088.s005.docx]
