# Supplementary material for: Estimation of D-Arabinose by Gas Chromatography/Mass Spectrometry as Surrogate for Mycobacterial Lipoarabinomannan in Human Urine
Source: PLoS One. 2015 Dec 3;10(12):e0144088. doi: 10.1371/journal.pone.0144088 (PMC4669150; doi:10.1371/journal.pone.0144088)
Supplement: S1 Table — (DOCX) [file pone.0144088.s006.docx]

**Table S-1:** **FIND urine sample and 100 Blind FIND urine samples: D-Ara analysis in File S1**

| **Smear & Culture TB +ve Urine Samples** | | | | **Smear & Culture TB -ve Urine Samples** | | | |
| --- | --- | --- | --- | --- | --- | --- | --- |
| **Sample ID** | **Other Infection** | **Calc. LAM (ng/mL)** | **Sample ID** | **Other Infection** | | **Calc. LAM (ng/mL)** | |
| **K102** |  | **22.8** ^a^ **/20.2** ^b^ | **K69** | **HIV, atyp/ pneu** | | **0.0** ^a^ **/0.0** ^c^ | |
| **K130** |  | **17.8** ^a^ **/37.5** ^b^ | **K72** | **atyp/pneu** | | **0.0** ^a^ **/0.0** ^c^ | |
| **K133** |  | **16.7** ^a^ **/17.8** ^c^ | **K73** | **atyp/pneu** | | **0.0** ^a^ **/0.0** ^c^ | |
| **K137** |  | **12.1** ^c^ **/12.1** ^c^ | **K75** |  | | **0.0** ^a^ **/0.0** ^c^ | |
| **K15** |  | **38.0** ^a^ **/35.7** ^a^ | **K100** |  | | **0.0** ^a^ **/0.0** ^c^ | |
| **K53** |  | **19.1** ^c^**/20.6** ^c^ | **K101** | **atyp/pneu** | | **0.0** ^a^ **/0.0** ^c^ | |
| **K43** |  | **20.0** ^c^ **/27.3** ^b^ | **K108** | **atyp/pneu** | | **7.8** ^a^ **/7.8** ^c^ | |
| **K188** |  | **17.1** ^c^ **/20.8** ^b^ | **K145** | **atyp/pneu** | | **0.0** ^a^ **/0.0** ^c^ | |
| **K66** |  | **8.4** ^a^ **/32.1** ^c^ | **K155** | **atyp/pneu** | | **0.0** ^a^ **/0.0** ^c^ | |
| **K139** |  | **17.8** ^c^ **/21.7** ^c^ | **K175** | **atyp/pneu** | | **0.0** ^a^ **/0.0** ^c^ | |
| **K71** | **HIV** | **37.5** ^a^ **/47.6** ^b^ | **K209** | **NK** | | **0.0** ^a^ **/0.0** ^c^ | |
| **K76** |  | **29.7** ^a^ **/45.2** ^b^ | **K87** | **atyp/pneu** | | **23.8** ^a^ **/25.6** ^c^ | |
| **K77** |  | **17.3** ^a^ **/41.0** ^b^ | **K119** | **atyp/pneu** | | **12.6** ^a^ **/14.2** ^c^ | |
| **K78** |  | **21.7** ^a^ **/19.0** ^c^ | **K132** | **atyp/pneu** | | **0.0** ^a^ **/0.0** ^c^ | |
| **K79** |  | **15.7** ^c^ **/18.0** ^a^ | **K140** | **atyp/pneu** | | **0.0** ^a^ **/0.0** ^c^ | |
| **K11** |  | **0.0** ^a^ **/0.0** ^a^ | **K106** | **other** | | **0.0** ^a^ | |
| **K57** |  | **0.0** ^a^ **/0.0** ^a^ | **K107** | **atyp/pneu** | | **0.0** ^a^ | |
| **K95** | **HIV** | **0.0** ^a^ **/0.0** ^a^ | **K112** |  | | **0.0** ^a^ | |
| **K23** | **HIV** | **0.0** ^a^ **/0.0** ^a^ | **K114** | **Pneumonia** | | **0.0** ^a^ | |
| **K138** | **HIV** | **0.0** ^a^ **/0.0**^a^ | **K118** |  | | **0.0** ^a^ | |
| **K195** | **HIV** | **0.0** ^a^ **/0.0**^a^ | **K121** | **other** | | **0.0** ^a^ | |
| **K135** | **HIV** | **0.0** ^a^ **/0.0^a^** | **K122** |  | | **0.0** ^a^ | |
| **K80** |  | **4.4^a^** | **K123** | **other** | | **0.0** ^a^ | |
| **K25** |  | **6.0 ^a^** | **K126** |  | | **0.0** ^a^ | |
| **K32** |  | **6.0 ^a^** | **K127** | **other** | | **0.0** ^a^ | |
| **K156** |  | **6.3 ^a^** | **K129** | **atyp/pneu** | | **0.0** ^a^ | |
| **K19** |  | **6.7 ^a^** | **K136** | **Pneumonia** | | **0.0** ^a^ | |
| **K7** |  | **7.1 ^a^** | **K146** | **Pneumonia** | | **0.0** ^a^ | |
| **K59** |  | **8.2 ^a^** | **K148** | **atyp/pneu** | | **0.0** ^a^ | |
| **K67** |  | **8.5 ^a^** | **K149** | **other** | | **0.0** ^a^ | |
| **K61** |  | **9.0 ^a^** | **K150** |  | | **0.0** ^a^ | |
| **K128** |  | **9.5 ^a^** | **K158** |  | | **0.0** ^a^ | |
| **K18** |  | **9.5 ^a^** | **K159** |  | | **0.0** ^a^ | |
| **K142** |  | **9.5 ^a^** | **K16** | **atyp/pneu** | | **0.0** ^a^ | |
| **K153** |  | **9.5 ^a^** | **K160** | **Pneumonia** | | **0.0** ^a^ | |
| **K34** | **HIV** | **9.5 ^a^** | **K164** | **Pneumonia** | | **0.0** ^a^ | |
| **K51** |  | **10.7 ^a^** | **K166** | **HIV** | | **0.0** ^a^ | |
| **K157** |  | **10.7 ^a^** | **K169** | **atyp/pneu** | | **0.0** ^a^ | |
| **K10** |  | **10.7 ^a^** | **K170** | **other** | | **0.0** ^a^ | |
| **K27** |  | **11.1 ^a^** | **K173** | **other** | | **0.0** ^a^ | |
| **K178** |  | **11.3 ^a^** | **K176** | **atyp/pneu** | | **0.0** ^a^ | |
| **K124** | **HIV** | **11.7 ^a^** | **K179** | **atyp/pneu** | | **0.0** ^a^ | |
| **K99** |  | **11.9 ^a^** | **K182** | **HIV, Pneumonia** | | **0.0** ^a^ | |
| **K22** |  | **11.9 ^a^** | **K185** | **atyp/pneu** | | **0.0** ^a^ | |
| **K152** |  | **11.9 ^a^** | **K186** | **atyp/pneu** | | **0.0** ^a^ | |
| **K30** |  | **11.9 ^a^** | **K187** | **pneumonia** | | **0.0** ^a^ | |
| **K172** |  | **11.9 ^a^** | **K189** | **other** | | **0.0** ^a^ | |
| **K165** |  | **11.9 ^a^** | **K191** | **other** | | **0.0** ^a^ | |
| **K117** |  | **13.0 ^a^** | **K193** |  | | **0.0** ^a^ | |
| **K48** |  | **14.2 ^a^** | **K197** |  | | **0.0** ^a^ | |
| **K86** |  | **14.3 ^a^** | **K198** | **atyp/pneu** | | **0.0** ^a^ | |
| **K134** |  | **14.3 ^a^** | **K21** | **atyp/pneu** | | **0.0** ^a^ | |
| **Smear & Culture TB +ve Urine Samples** | | | | **Smear & Culture TB -ve Urine Samples** | | | |
| **Sample ID** | **Other Infection** | **Calc. LAM (ng/mL)** | **Sample ID** | **Other Infection** | | **Calc. LAM (ng/mL)** | |
| **K9** |  | **14.3 ^a^** | **K24** | **other** | | **0.0** ^a^ | |
| **K200** |  | **14.3 ^a^** | **K28** |  | | **0.0** ^a^ | |
| **K92** |  | **15.0 ^a^** | **K31** | **other** | | **0.0** ^a^ | |
| **K168** | **HIV** | **15.4 ^a^** | **K35** | **atyp/pneu** | | **0.0** ^a^ | |
| **K96** |  | **16.1 ^a^** | **K36** |  | | **0.0** ^a^ | |
| **K190** | **HIV** | **16.1 ^a^** | **K38** | **atyp/pneu** | | **0.0** ^a^ | |
| **K50** |  | **16.6 ^a^** | **K39** |  | | **0.0** ^a^ | |
| **K183** |  | **16.7 ^a^** | **K4** | **HIV** | | **0.0** ^a^ | |
| **K115** |  | **16.7 ^a^** | **K41** | **atyp/pneu** | | **0.0** ^a^ | |
| **K171** |  | **16.7 ^a^** | **K42** |  | | **0.0** ^a^ | |
| **K194** |  | **16.7 ^a^** | **K43** | **HIV, pneumonia** | | **0.0** ^a^ | |
| **K167** |  | **16.7 ^a^** | **K44** | **other** | | **0.0** ^a^ | |
| **K33** |  | **17.8 ^a^** | **K46** | **pneumonia** | | **0.0** ^a^ | |
| **K91** |  | **17.8 ^a^** | **K52** |  | | **0.0** ^a^ | |
| **K141** |  | **19.0 ^a^** | **K55** |  | | **0.0** ^a^ | |
| **K177** |  | **19.0 ^a^** | **K56** | **pneumonia** | | **0.0** ^a^ | |
| **K84** |  | **19.0 ^a^** | **K58** | **HIV** | | **0.0** ^a^ | |
| **K103** | **HIV** | **19.0 ^a^** | **K62** | **other** | | **0.0** ^a^ | |
| **K154** |  | **19.8 ^a^** | **K63** | **other** | | **0.0** ^a^ | |
| **K94** |  | **20.0 ^a^** | **K64** | **other** | | **0.0** ^a^ | |
| **K13** |  | **20.2 ^a^** | **K65** | **other** | | **0.0** ^a^ | |
| **K110** | **HIV** | **21.3 ^a^** | **K8** |  | | **0.0** ^a^ | |
| **K12** |  | **21.4 ^a^** | **K88** |  | | **0.0** ^a^ | |
| **K113** |  | **21.4 ^a^** | **K97** | **atyp/pneu** | | **0.0** ^a^ | |
| **K162** |  | **21.4 ^a^** | **K98** |  | | **0.0** ^a^ | |
| **K90** |  | **21.4 ^a^** | **K60** | **HIV** | | **10.8^a^** | |
| **K199** |  | **22.2 ^a^** | **K181** | **atyp/pneu** | | **14.3 ^a^** | |
| **K192** |  | **23.8 ^a^** | **K40** | **HIV** | | **14.3 ^a^** | |
| **K47** |  | **23.8 ^a^** | **K93** | **HIV** | | **16.7 ^a^** | |
| **K3** |  | **25.0 ^a^** | **K5** |  | | **18.5 ^a^** | |
| **K14** |  | **25.0 ^a^** | **K116** | **pneumonia** | | **20.2 ^a^** | |
| **F29** | **HIV** | **25.2 ^a^** | **K161** | **HIV** | | **31.0 ^a^** | |
| **K54** | **HIV** | **25.9 ^a^** | **K89** | **atyp/pneu** | | **31.4 ^a^** | |
| **K131** |  | **26.8 ^a^** |  |  |  | |  |
| **K125** | **HIV** | **26.9 ^a^** | *^a^* 1/5 of 3.5 mL sample analyzed, *^b^* 2/5 of 3.5 mL sample analyzed, ^c^ 1/3 of 3.5 mL sample analyzed;  NK= not Known, ND = not determined | | | | |
| **K26** |  | **30.9 ^a^** |  |  |  |  |  |
| **K105** | **HIV** | **32.2 ^a^** |  |  |  |  |  |
| **K163** |  | **33.3 ^a^** |  |  |  |  |  |
| **K68** |  | **40.0 ^a^** |  |  |  |  |  |
| **K20** |  | **40.4 ^a^** |  |  |  |  |  |
| **K109** |  | **42.8 ^a^** |  |  |  |  |  |

**100 Blind FIND urine samples**

| **TB Sputum smear & culture negative** | | | **TB Sputum smear & culture positive** | | |
| --- | --- | --- | --- | --- | --- |
| **CSUID** | **LAM (ng/mL)^a/b^** | **Other infection** | **CSUID** | **LAM (ng/mL)^a/b^** | **Other infection** |
| **B15** | **32.1^a^/35.7^b^** | **TB likely** | **B10** | **30.0^b^/28.7^b^** |  |
| **B23** | **29.7^a^/33.9^b^** | **Pneu/atyp** | **B11** | **45.2^b^/51.7^b^** |  |
| **B24** | **16.6^a^/18.0^b^** | **Pneu/atyp** | **B13** | **44.0^a^/40.4^b^** |  |
| **B26** | **0.0^a^/0.0^b^** | **Pneumonia** | **B17** | **47.2^b^/44.6^b^** |  |
| **B3** | **19.6^b^/21.4^b^** | **Pneumonia** | **B25** | **8.6^b^/11.2^b^** |  |
| **B37** | **0.0^a^/0.0^b^** | **Pneumonia** | **B27** | **27.3^a^/31.3^b^** |  |
| **B60** | **0.0^a^/0.0^b^** | **Pneu/atyp** | **B29** | **39.8^b^/30.9^b^** |  |
| **B63** | **0.0^a^/0.0^b^** | **Pneu/atyp** | **B34** | **17.8^a^/16.6^b^** |  |
| **B79** | **18.5^a^/16.6^b^** | **Pneu/atyp** | **B35** | **25.0^a^/26.1^b^** |  |
| **B93** | **0.0^a^/0.0^b^** |  | **B38** | **42.8^a^/35.7^b^** |  |
| **B12** | **21.4^a^/20.9^b^** | **HIV, Pneu/atyp** | **B66** | **64.3^b^/57.8^b^** |  |
| **B2** | **13.5^a^/23.8^b^** | **HIV, Pneu/atyp** | **B73** | **19.0^a^/25.5^b^** |  |
| **B30** | **0.0^a^/0.0^b^** | **HIV** | **B1** | **47.6^a^/47.8^b^** |  |
| **B31** | **0.0^a^/0.0^b^** | **HIV** | **B16** | **14.8^b^/14.0^b^** | **HIV** |
| **B36** | **0.0^a^/0.0^b^** | **HIV** | **B21** | **68.4^b^/71.4^b^** | **HIV** |
| **B5** | **19.0^a^/21.4^b^** | **HIV, Pneu/atyp** | **B32** | **34.5^a^/32.4^b^** | **HIV** |
| **B64** | **39.2^b^/43.4^b^** | **HIV, Pneu/atyp** | **B4** | **19.0^a^/28.5^b^** | **HIV** |
| **B68** | **0.0^a^/0.0^b^** | **HIV, Pneu/atyp** | **B7** | **36.9^a^/28.5^b^** | **HIV** |
| **B91** | **0.0^a^/0.0^b^** | **HIV, Pneu/atyp** | **B72** | **36.6^a^/34.4^b^** | **HIV** |
| **B28** | **0.0^a^/0.0^b^** | **Other** | **B9** | **35.0^a^/44.0^b^** | **HIV** |
| **B33** | **0.0^a^/0.0^b^** | **HIV, Other** | **B18** | **16.6^a^/20.0^b^** | **Pneu/atyp** |
| **B41** | **0.0^a^/0.0^b^** | **Pneu/atyp** | **B19** | **27.8^a^/25.1^a^** | **HIV** |
| **B6** | **29.7^a^/38.0^b^** | **HIV, Other** | **B20** | **0.0^a^/0.0^b^** | **HIV** |
| **B14** | **26.6^a^/28.5^b^** | **HIV** | **B22** | **27.3^a^/28.0^a^** |  |
| **B105** | **0.0^a^** | **HIV** | **B46** | **23.8^a^/19.8^b^** | **HIV** |
| **B103** | **0.0 ^a^** | **HIV** | **B8** | **27.3^a^/30.0^b^** | **HIV** |
| **B134** | **25.0 ^a^** | **HIV** | **B142** | **6.9 ^a^** |  |
| **B138** | **0.0 ^a^** | **HIV** | **B146** | **4.8 ^a^** |  |
| **B101** | **0.0 ^a^** | **HIV** | **B127** | **37.8 ^a^** | **HIV** |
| **B106** | **0.0 ^a^** | **HIV** | **B148** | **7.8 ^a^** |  |
| **B143** | **0.0 ^a^** |  | **B122** | **65.8 ^a^** | **HIV** |
| **B102** | **0.0 ^a^** |  | **B132** | **11.4 ^a^** | **HIV** |
| **B139** | **0.0 ^a^** | **HIV** | **B144** | **18.1 ^a^** |  |
| **B119** | **0.0 ^a^** | **HIV, Pneu/atyp** | **B108** | **5.4 ^a^** |  |
| **B104** | **0.0 ^a^** | **HIV** | **B145** | **14.2 ^a^** |  |
| **B123** | **47.6 ^a^** | **HIV** | **B136** | **4.8 ^a^** |  |
| **B126** | **0.0 ^a^** |  | **B110** | **8.7 ^a^** | **HIV** |
| **B150** | **48.7 ^a^** | **HIV, Pneu/atyp** | **B135** | **7.8 ^a^** | **HIV** |
| **B149** | **0.0 ^a^** | **HIV** | **B107** | **7.2 ^a^** | **HIV** |
| **B116** | **34.6 ^a^** |  | **B128** | **47.8 ^a^** | **HIV** |
| **B124** | **0.0 ^a^** |  | **B118** | **32.6 ^a^** | **HIV** |
| **B114** | **0.0 ^a^** | **Pneumonia** | **B117** | **34.6 ^a^** | **HIV** |
| **B115** | **23.3 ^a^** | **Pneumonia** | **B121** | **70.0 ^a^** |  |
| **B125** | **30.0 ^a^** | **Pneumonia** | **B129** | **28.7 ^a^** | **HIV** |
| **B137** | **0.0 ^a^** | **Pneu/atyp** | **B112** | **31.6 ^a^** |  |
| **B133** | **0.0 ^a^** | **Pneumonia** | **B111** | **27.9 ^a^** |  |
| **B130** | **0.0 ^a^** | **Pneu/atyp** | **B109** | **4.8 ^a^** | **HIV** |
| **B140** | **0.0 ^a^** | **Pneumonia** | **B120** | **21.8 ^a^** |  |
| **B141** | **54.1 ^a^** | **other** | **B147** | **24.6 ^a^** | **HIV** |
| *^a^* 1/5 of 3.5 mL sample analyzed,  *^b^* 2/5 of 3.5 mL sample analyzed | | | **B113** | **25.0 ^a^** |  |
|  |  |  | **B131** | **20.0 ^a^** | **HIV** |
